# Patients referred to but not taken on to an outpatient parenteral antimicrobial therapy (OPAT) service: the impact of infection specialist advice on the assessment and clinical outcomes

**DOI:** 10.1093/jacamr/dlag090

**Published:** 2026-06-03

**Authors:** Mary Rimbi, Simon Pybus, Sophie Dodds, Beth White, Neil Ritchie, R Andrew Seaton

**Affiliations:** Infectious Diseases Department, Queen Elizabeth University Hospital, 1345 Govan Road, Glasgow G51 4TF, UK; Infectious Diseases Department, Queen Elizabeth University Hospital, 1345 Govan Road, Glasgow G51 4TF, UK; Infectious Diseases Department, Queen Elizabeth University Hospital, 1345 Govan Road, Glasgow G51 4TF, UK; Infectious Diseases Department, Queen Elizabeth University Hospital, 1345 Govan Road, Glasgow G51 4TF, UK; Infectious Diseases Department, Queen Elizabeth University Hospital, 1345 Govan Road, Glasgow G51 4TF, UK; Infectious Diseases Department, Queen Elizabeth University Hospital, 1345 Govan Road, Glasgow G51 4TF, UK

## Abstract

**Objectives:**

This study explored antimicrobial stewardship opportunities, clinical impact and patient outcomes in those referred to an outpatient parenteral antimicrobial therapy (OPAT) service, screened by an infection specialist but not taken on in a large tertiary centre.

**Methods:**

All OPAT referrals that were screened but not taken on by the NHS Greater Glasgow and Clyde OPAT service over a 3 year period (2022–2024) were reviewed. Those that were advised an oral antibiotic option that did not require close monitoring and did not require specialist OPAT follow-up were analysed for reason for referral, duration of antibiotic prescribed and patient outcome (readmission and mortality) at 30 days.

**Results:**

During the study period 5024 patients were referred to OPAT and 1467 (29.2%) were not taken onto the service. Of these 517 (35%) were given advice to switch to an oral antibiotic option that did not require specific OPAT monitoring. These included referrals for bone and joint, complex intra-abdominal and *Staphylococcus aureus* bacteraemia or other bloodstream infections, amongst others. ‘Oral recommended’ resulted in a total of 13 909 days of oral therapy at home over 3 years. Thirty-day mortality was 3.9% (20/517) and 30 day readmission directly as a result of the infection or the treatment was 6.4% (32/498).

**Conclusions:**

These data highlight the central role of antimicrobial stewardship in OPAT gate keeping and the importance of measuring the unseen and wider healthcare service impact of OPAT. Assuming those referred had remained on IV therapy then the equivalent of 13 909 days of inpatient or OPAT IV days was avoided.

## Introduction

Outpatient parenteral antimicrobial therapy (OPAT) is a safe, clinically effective and cost-effective standard of care for many infections otherwise requiring inpatient care.^1^ OPAT facilitates early discharge or admission avoidance for medically stable patients requiring IV antimicrobial therapy.^2^ Complex outpatient antimicrobial therapy (COpAT), which describes a mixed model service incorporating both complex oral and IV antimicrobial therapy,^3^ has become a fundamental part of modern OPAT practice, acknowledging appropriate oral therapy to be non-inferior to IV therapy in many infectious syndromes.^4^ This development has largely been influenced by landmark papers that have increased confidence in early oral switch for the management of endocarditis, *Staphylococcus aureus* bacteraemia, and bone and joint infections.^5–7^

Clinical outcomes following OPAT for a range of infections are well described, with more than 90% partial or complete success observed in the large BSAC OPAT registry study.^2^ OPAT has also been shown to be highly cost-effective, with the most frequently treated conditions managed at a fraction of the cost of inpatient care.^8^ Whilst such metrics are crucial to support funding and expansion of OPAT services, an as yet unmeasured function of OPAT is the impact of infection specialist/antimicrobial stewardship (AMS) advice on the assessment and clinical outcomes of those patients referred but then not taken on to the OPAT service. Following the observation that many patients were referred to our OPAT service but following specialist assessment required neither IV nor close specialist monitoring via OPAT, we set out to explore the clinical impact and patient outcomes in this group.

## Patients and methods

The NHS Greater Glasgow and Clyde health board (NHS GGC) OPAT service was established in 2000 and is a standalone service that has expanded to cover an adult population of approximately 1.3 million persons.^9^ Referrals are received from primary, secondary and tertiary care from a wide range of clinical specialties across four acute hospital sites. The NHS GGC OPAT service is situated in the Queen Elizabeth University Hospital but outreaches to all hospitals within the health board.

Referrals to OPAT are made and recorded electronically via the patient management system Trakcare. Referrals are screened and vetted at least 5 days per week by an infectious disease specialist to determine appropriateness of referral and to design the most appropriate ongoing infection management plan. Following assessment of the referral and associated clinical data (radiological, microbiological, other laboratory variables and consideration of comorbidities and co-medication) referrers are either contacted to clarify further clinical details, the patient is physically reviewed, or a recommendation is proposed remotely for ongoing infection management. Those judged to be appropriate for OPAT are assigned to either IV or oral antimicrobial therapy with a plan for appropriate monitoring overseen via an electronic virtual ward platform. Patients assigned to outpatient (OP) IV therapy are reviewed by an OPAT clinical nurse specialist to determine their suitability for either self- or carer-administration, continuous infusion therapy or clinic attendance. For those advised to be discharged on complex oral therapy, a patient information leaflet and counselling is provided, and an appointment for review by the OPAT nurse specialist is arranged following discharge. Complex oral therapy refers to drugs such as co-trimoxazole, fluoroquinolones, linezolid and clindamycin that require toxicity monitoring. Potential drug interactions/contraindications are assessed by an antimicrobial pharmacist. Patients taken on to OPAT are then managed and followed in alignment with the BSAC OPAT Good Practice Recommendations.^1^ Although the IDSA recommends that serial laboratory testing should be monitored in patients receiving OPAT, there are no recommendations on the frequency of outpatient follow-up.^10^

Those patients judged to be not suitable or appropriate for OPAT are assigned a reason on the electronic patient vetting system and a contemporaneous clinical note is recorded on the electronic patient record, which is communicated to the referrer. Reasons for not taking on to OPAT include, but are not restricted to, ongoing inpatient care recommended, no suitable OPAT regimen available, lack of service capacity (e.g. lack of appointments for antibiotic administration, lack of staff to train patients/family members, lack of isometric volume pumps to facilitate continuous infusions), recommendation to stop treatment, and oral therapy recommended with no specific OPAT monitoring requirements (NOPAT). For those where ‘NOPAT’ is recommended, a suggestion may be made for follow-up by the referring specialist or specific follow-up in an infectious diseases clinic if appropriate.

Referrals to the NHS GGC OPAT service were retrospectively reviewed over a 3 year period (2022–2024). Those patients who were screened and not taken on to OPAT were identified, and data were further interrogated for those for whom NOPAT was advised. Patient-identifiable data were anonymized. Data were collated on reason for referral, oral antibiotic recommended, recommended duration of antibiotic prescription, and patient outcome including readmission and mortality at 30 days post recommendation for oral therapy. For those patients who were readmitted, the reason for readmission (infection related or unrelated) was recorded.

Data were collected as part of routine clinical care. Ethical approval was not required.

## Results

### Referrals to OPAT

The NHS GGC OPAT service received a total of 5024 referrals between January 2022 and December 2024. Of these 3557 received either OP IV therapy or complex oral therapy and are not considered further here. A total of 1467 (29.2%) were not taken onto the service (Table 1). Most frequent reasons for not being taken on to OPAT were antimicrobial stewardship interventions, i.e. oral therapy recommended or antibiotics recommended to stop (*n* = 638), and patients judged to be not suitable for the service (*n* = 510). Only a small number of patients could not access OPAT due to lack of availability of an appropriate OPAT regimen or lack of capacity within the team. Excluding administrative reasons for not taking on to OPAT (*n* = 201), such as duplicate referral or incomplete clinical information, the most frequent single reason for not taking on to OPAT was oral antimicrobial therapy recommended without OPAT follow-up (*n* = 517/1266, 40.8%).

**Table 1.** Reasons for not taking onto OPAT services (NOPAT)

Antimicrobial stewardship	Oral recommended Antibiotics not required	517 121
Refer to alternative service	Non-GGC OPAT Hospital-at-home service	70 1
Patient not suitable	Patient medical (non-infection) circumstances not suitable Patient/carer refused Patient social circumstances not suitable Infection requires inpatient management (e.g. surgical source control, antimicrobial regimen that could not be feasibly delivered in the OPAT setting)	208 89 35 178
Administrative	Duplicate referral Referral withdrawn Insufficient clinical information	106 50 45
Other	Antibiotic regime not suitable Hospital OPAT team no capacity	22 19
	Missing data	6
Total		1467

### Indications for oral therapy recommended and no OPAT follow-up

Oral therapy with no OPAT follow-up was recommended most frequently in bone and joint (238; 46%), complicated intra-abdominal (132; 25.5%), endovascular and bloodstream (37; 7.1%), urinary tract (27; 5.2%), respiratory (20; 3.9%), skin and soft tissue (17; 3.3%), and endocarditis (9; 1.7%) infections. As a rule, complex infections were routinely followed up in infectious disease consultant clinics or specialist surgical clinics.

Antibiotics recommended in the oral antimicrobial therapy group for the most frequently managed groups of infections are listed in Table 2. It should be noted that only patients who had completed toxicity monitoring for COpAT regimens (such as co-trimoxazole, fluoroquinolones, linezolid and clindamycin) as inpatients are included. Those who had not completed toxicity monitoring prior to discharge were managed under COpAT and are not discussed in this article.

**Table 2.** Most common antibiotic regimens used

Infection	Antibiotics used	*n*	Median (Q1–Q3) days of treatment, including IV prior to NOPAT
Bone and joint infection (*n* = 238)	Doxycycline Clindamycin Co-trimoxazole Amoxicillin with co-amoxiclav Ciprofloxacin Other	63 49 20 18 17 71	42 (42–42)
Skin and soft tissue infection (*n* = 17)	Clindamycin Flucloxacillin Doxycycline Other	4 3 2 8	14 (10–14)
Intra-abdominal infection (*n* = 132)	Amoxicillin with co-amoxiclav Ciprofloxacin Ciprofloxacin and metronidazole Levofloxacin and metronidazole Co-amoxiclav Other	73 11 9 9 6 24	42 (28–42)
Endocarditis (*n* = 9)	Amoxicillin Clindamycin Other	4 3 2	42 (42–42)
Endovascular infections (*n* = 16)	Clindamycin Other	8 8	42 (28–42)
Respiratory tract infections (*n* = 20)	Amoxicillin and co-amoxiclav Levofloxacin Co-trimoxazole Co-amoxiclav Other	5 3 2 2 8	28 (14–42)
*Staphylococcus aureus* bacteraemia (*n* = 7)	Clindamycin Co-trimoxazole Other	2 2 3	14 (10.5–21)
Candidaemia (*n* = 2)	Fluconazole	2	14 (14–14)
Other bacteraemia (*n* = 12)	Clindamycin Ciprofloxacin Other	3 3 6	14 (8–38.5)
Urinary tract infection (*n* = 27)	Ciprofloxacin Cefalexin Fosfomycin Linezolid Co-amoxiclav Other	8 7 3 2 2 5	7 (6.5–7)

‘Other’ represents a range of options that were selected for one patient

‘Q1’ refers to first quartile. ‘Q3’ refers to third quartile. Oral β-lactams (*n* = 119), clindamycin (*n* = 69) and doxycycline (*n* = 65) were the most commonly used oral agents in the cohort. Where fluoroquinolones, linezolid, clindamycin or co-trimoxazole are listed, any required toxicity monitoring had already occurred as an inpatient.

Our standard dosing was co-amoxiclav 625 mg plus amoxicillin 500 mg q8h; clindamycin 600 mg q8h if BMI <30; clindamycin 900 mg q8h if BMI >30; amoxicillin 1 g q6h.

**Figure 1.**
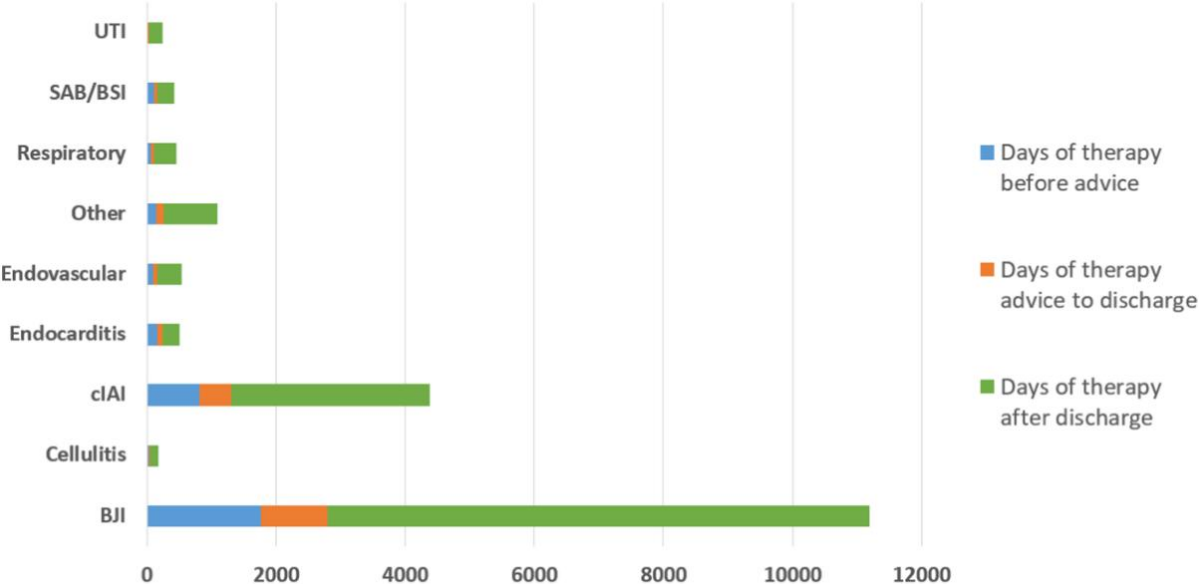
Days of therapy before advice given, between advice and discharge, and after discharge by indication in ‘Oral recommended—no OPAT follow-up’ group over 3 year period (2022–2024). BJI, bone and joint infections; BSI, bloodstream infections; cIAI, complex intra-abdominal infections; SAB, *Staphylococcus aureus* bacteraemia; UTI, urinary tract infections.

Complete data for recommended duration of treatment in the oral antimicrobial group were available for 490 patients. Oral therapy recommended without OPAT follow-up resulted in a total of 13 909 days of oral antimicrobial therapy at home over the 3 year period (Figure 1).

### Patient outcomes

Readmission at 30 days from the date when oral antibiotic advice was given was available for 498 patients. Eighty-six (17.2%) were readmitted, and the majority of these (74, 86%) were unplanned. Overall, 32 (6.4%) patients who were discharged following oral therapy recommendations were readmitted at 30 days directly as a result of the infection or the treatment they were referred for. Readmission occurred in 2 (5%) of those with endovascular infection or bloodstream infection, 17 (7%) of those with bone and joint infection, 9 (7%) in those with intra-abdominal infection, 3 (11%) of those with urinary tract infection (UTI), and 1 being treated for necrotizing otitis externa. No patients being managed for endocarditis were readmitted within 30 days.

Amongst the ‘oral recommended’ group, 30 day mortality was 3.9% (20/517). Cause of death was related to the infection referred to OPAT in three patients (one respiratory, one intra-abdominal and one skin soft tissue). Cause of death was malignancy in five, and cause of death was unknown or non-infection-related in the remainder. Excluding those where death was due to underlying malignancy, mortality was estimated to be 2.9%. Most deaths for which cause of death was unknown occurred in the community, and community medical certificates of death were not available on our system. It was not possible to determine the influence of comorbidity on mortality or whether mortality was expected or unexpected.

## Discussion

OPAT services facilitate early discharge (or in some cases complete avoidance of admission) for patients with infection who require treatment with either IV antibiotics or complex oral antibiotic regimens that require monitoring.

Assessment of the OPAT referral is a key antimicrobial stewardship opportunity, where antibiotics can be stopped if deemed not indicated, early intravenous to oral switch of therapy (IVOST) encouraged, or a change to antibiotics with a safer side effect profile for which OPAT/COpAT monitoring is not required. These referrals, in which stewardship considerations are foremost, are often listed as ‘rejected’ and hence data on clinical impact are not captured within normal OPAT activity.

We have shown that a high proportion of patients referred to OPAT and assessed by an infection specialist were recommended to switch to oral antimicrobial therapy without specific OPAT monitoring. Assuming the recommendations to complete the recommended course of oral antimicrobials had been followed, up to 38 years of inpatient or OPAT IV or oral monitoring days were potentially avoided by this stewardship intervention. Switching to oral antibiotics not only reduces length of stay and improves patient convenience but also has positive environmental impact due to reduction in travel time for review and consumables required to deliver IV therapy.

We attempted to quantify any negative impact of this intervention. Readmission due to the infection or treatment following clinical advice to switch to oral therapy without OPAT follow-up was 6.2%, which compares favourably with most published experience for patients managed within a traditional OPAT service.^11^ All-cause mortality at 30 days from the date of advice was 3.9%, reducing to 2.9% when deaths known to be secondary to malignancy were excluded. It was not possible to determine how many of the remaining patients died directly as a consequence of the infection because the cause of death in the community could not be determined. Deaths directly attributable to infection may therefore be lower than 2.9%. There were no studies identified that were directly comparable, although in a 20 year study of post discharge mortality following emergency general surgical admission in NHS Scotland, 30 day mortality (from admission) was 3.8%, increasing to 6.4% at 90 days.^12^ Mortality following a course of OPAT is dependent on the condition being treated and underlying comorbidity. In a meta-analysis of outcomes following OPAT management of endocarditis, mortality following OPAT was estimated to be between 3% and 8%.^13^ Attributable mortality in bone and joint/orthopaedic infection following OPAT would be expected to be lower. Patients with diabetic foot infection are at higher risk of infection relapse as well as mortality due to other complications of diabetes. Infection relapse may be expected to be higher in the population of patients where source control had not been possible due to frailty or associated operative risks. We were unable to assess the rate of unplanned antibiotic discontinuation and did not assess the extent to which advice given was followed by referrers. Both may have influenced readmission and mortality rates.

This review highlights the positive clinical service impact of infection specialist OPAT referral assessment and triage, and shows the central importance of antimicrobial stewardship within the OPAT assessment process. OPAT/CoPAT provides infection specialist follow-up and monitoring of patients with complex infections treated with either IV or oral antimicrobial therapy and provides opportunity to escalate, de-escalate or switch therapy in the community when required.^3^ Further work is required to identify potentially higher risk patient groups with complex infections in whom traditional OPAT monitoring is not deemed necessary but who may benefit from more intensive follow-up via OPAT. These data serve as a reminder that OPAT assessment/vetting is a dynamic and core part of the OPAT service.
